# Supplementary material for: Proteomic-Based Analysis of Hypoxia- and Physioxia-Responsive Proteins and Pathways in Diffuse Large B-Cell Lymphoma
Source: Cells. 2021 Aug 8;10(8):2025. doi: 10.3390/cells10082025 (PMC8392495; doi:10.3390/cells10082025)
Supplement: Supplementary file 1 [file cells-10-02025-s001.zip › Cells, supplementarry Figures.pdf]

cells

MDPI

Article

Proteomic-Based Analysis of Hypoxia- and Physioxia-Responsive Proteins and Pathways in Diffuse Large B-Cell Lymphoma

Kamila Duś-Szachniewicz , Katarzyna Gdesz- Birula , Krzysztof Zduniak , and Jacek R. Wiśniewski

Supplementary Figures

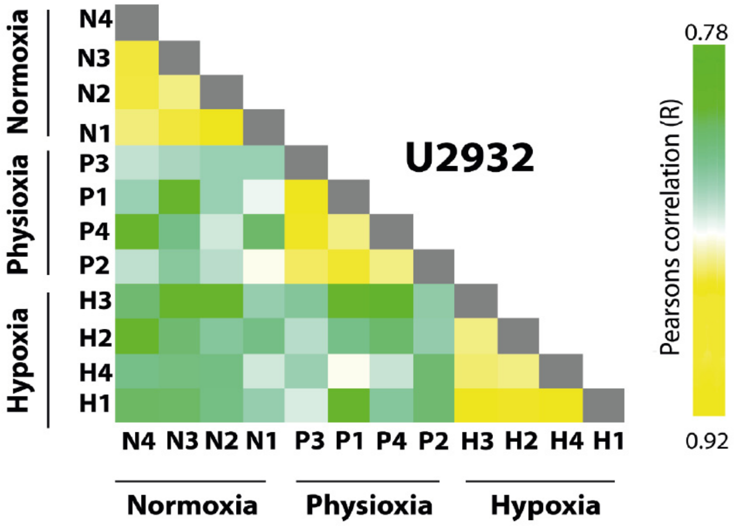

Figure S1. The heatmaps of the correlation coefficients of protein intensities after normalization showing reproducibility between biological replicates of U2932 proteomes under hypoxia (H, 1% of O_2), physioxia (P, 5% of O_2), and normoxia (N, 21% of O_2). Scale bar represents the range of the correlation coefficients (R) displayed. .

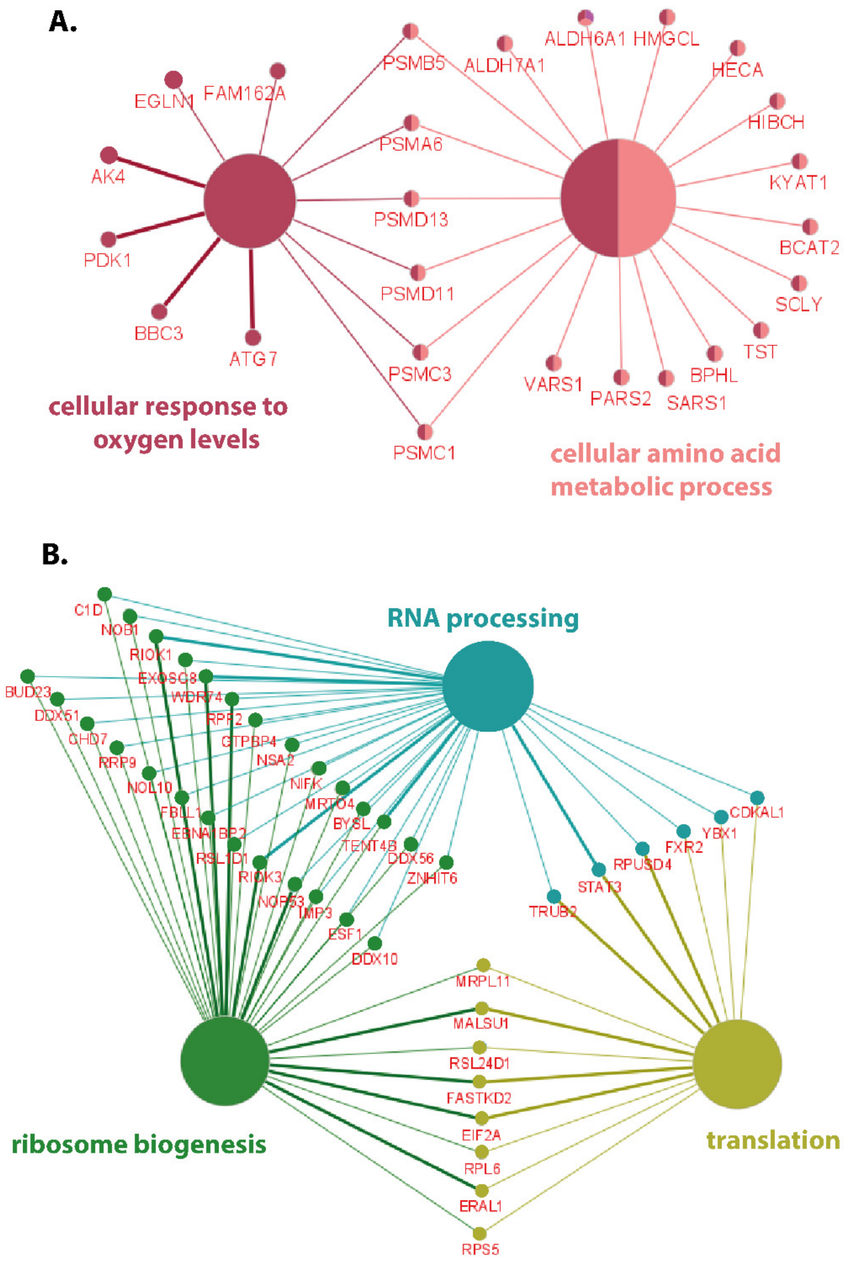

Figure S2. Cytoscape based ClueGo/CluePedia pathway visualization of (**A**) up-regulated, and (**B**) down-regulated proteins in Ri-1 cell line. Enriched pathways were obtained from the Gene Ontology- Biological Processes database. The size of the nodes corresponds to the significance of the GO pathway.

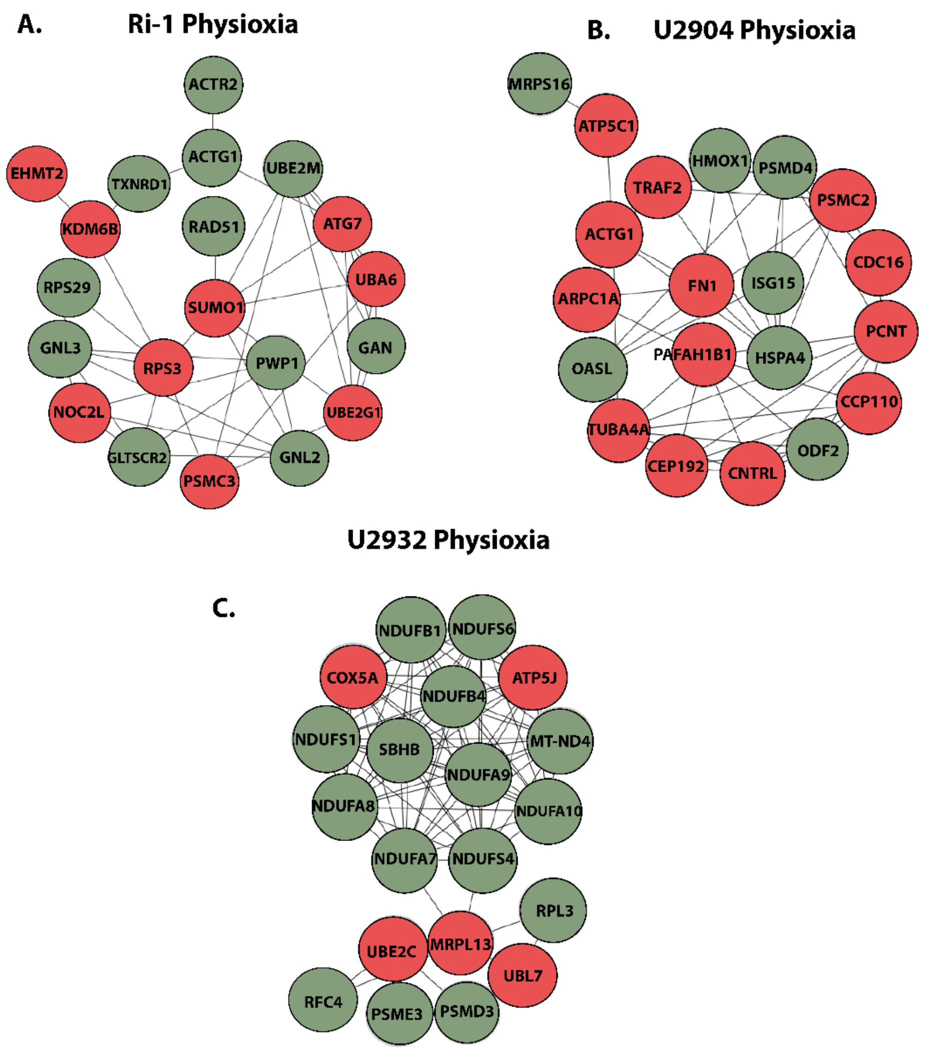

Figure S3. Top 20 hub proteins identified in (**A**) Ri-1, (**B**) U2904, and (**C**) U2932 cell lines under physioxia (5% O_2) based on the highest Degree score. Analyzed and generated by cytoHubba App of Cytoscape. Red and green nodes represent up- and down-regulated proteins, respectively.
